# Co-Circulation and Evolution of Polioviruses and Species C Enteroviruses in a District of Madagascar

**DOI:** 10.1371/journal.ppat.0030191

**Published:** 2007-12-14

**Authors:** Mala Rakoto-Andrianarivelo, Sophie Guillot, Jane Iber, Jean Balanant, Bruno Blondel, Franck Riquet, Javier Martin, Olen Kew, Bakolalao Randriamanalina, Lalatiana Razafinimpiasa, Dominique Rousset, Francis Delpeyroux

**Affiliations:** 1 Unité de Virologie Médicale, Institut Pasteur de Madagascar, Antananarivo, Madagascar; 2 Département Infection et Epidémiologie, PTMMH, Institut Pasteur, Paris, France; 3 National Center for Infectious Diseases, Centers for Disease Control and Prevention, Atlanta, Georgia, United States of America; 4 Département de Virologie, Biologie des Virus Entériques, Institut Pasteur, Paris, France; 5 Division of Virology, National Institute for Biological Standards and Control, Potters Bar, Hertfordshire, United Kingdom; 6 Programme Elargi de la Vaccination, Ministère de la Santé, du Planning Familial et de la Protection Sociale, Antananarivo, Madagascar; 7 Direction Régionale de la Santé Atsimo Andrefana, Ministère de la Santé, du Planning Familial et de la Protection Sociale, Toliara, Madagascar; 8 Unité de Virologie, Centre Pasteur du Cameroun, Yaoundé, Cameroun; Pennsylvania State University, United States of America

## Abstract

Between October 2001 and April 2002**,** five cases of acute flaccid paralysis (AFP) associated with type 2 vaccine-derived polioviruses (VDPVs) were reported in the southern province of the Republic of Madagascar. To determine viral factors that favor the emergence of these pathogenic VDPVs, we analyzed in detail their genomic and phenotypic characteristics and compared them with co-circulating enteroviruses. These VDPVs appeared to belong to two independent recombinant lineages with sequences from the type 2 strain of the oral poliovaccine (OPV) in the 5′-half of the genome and sequences derived from unidentified species C enteroviruses (HEV-C) in the 3′-half. VDPV strains showed characteristics similar to those of wild neurovirulent viruses including neurovirulence in poliovirus-receptor transgenic mice. We looked for other VDPVs and for circulating enteroviruses in 316 stools collected from healthy children living in the small area where most of the AFP cases occurred. We found vaccine PVs, two VDPVs similar to those found in AFP cases, some echoviruses, and above all, many serotypes of coxsackie A viruses belonging to HEV-C, with substantial genetic diversity. Several coxsackie viruses A17 and A13 carried nucleotide sequences closely related to the 2C and the 3D^pol^ coding regions of the VDPVs, respectively. There was also evidence of multiple genetic recombination events among the HEV-C resulting in numerous recombinant genotypes. This indicates that co-circulation of HEV-C and OPV strains is associated with evolution by recombination, resulting in unexpectedly extensive viral diversity in small human populations in some tropical regions. This probably contributed to the emergence of recombinant VDPVs. These findings give further insight into viral ecosystems and the evolutionary processes that shape viral biodiversity.

## Author Summary

Following extensive vaccination campaigns using the attenuated oral polio vaccine, wild polioviruses remain endemic in only a few countries. Nevertheless, several poliomyelitis outbreaks associated with vaccine-derived polioviruses (VDPVs) were reported in different parts of the world in recent years, particularly in Madagascar in 2002. We analyzed the molecular characteristics of Madagascar VDPVs and compared them with those of co-circulating enteroviruses. These VDPVs appear to be recombinant viruses between vaccine polioviruses and human enteroviruses of species C (HEV-C) and to present phenotypic characteristics similar to those of wild polioviruses including pathogenicity. Similar VDPVs and other enteroviruses, including several HEV-C of different types, were found in the stools of healthy children living in neighboring villages to where most of the poliomyelitis cases occurred. Some HEV-Cs showed sequences closely related to those of VDPVs, indicating genetic recombination between these viruses and vaccine polioviruses. There was also evidence of multiple genetic recombination events among other HEV-C isolates resulting in numerous different genotypes. These findings indicate that co-circulation of HEV-C and vaccine polioviruses and their evolution by recombination results in unexpectedly extensive viral diversity, at least in some small human populations, probably contributing to the emergence of recombinant VDPVs. Results of this study give further insight into the world of viruses and their biodiversity.

## Introduction

Polioviruses (PVs), members of the *Enterovirus* genus in the *Picornaviridae* family, are major human pathogens causing the acute paralytic disease poliomyelitis. The human enteroviruses (HEV) are classified into five species, HEV-A to -D, and the PV species (PV-1 to −3). The species HEV-C includes several serotypes of coxsackie A virus, and segregates in the same phylogenetic cluster (cluster C) as the PV species [1].

Enteroviruses, including PVs, are small non-enveloped viruses with a positive-strand RNA genome about 7.5 kb long. The single large coding region of the genome is flanked by 5′- and 3′-UTR. The coding region is translated as a single polyprotein that is processed by viral proteases to yield the mature viral proteins including the capsid proteins VP1 to VP4 and non-structural proteins including proteases and the RNA-dependent RNA polymerase 3D^pol^.

The World Health Organization's program for global eradication of poliomyelitis is based on immunization with the oral PV vaccine (OPV). The attenuated OPV strains of the three PV serotypes (Sabin 1, 2, and 3) replicate in the gut of OPV recipients where they efficiently mimic natural infection and thereby induce type-specific humoral and mucosal immunity. This strategy has reduced the incidence of polio worldwide by over 99% since the start of the global eradication program in 1988, and has restricted wild PV circulation to countries in western and central Africa and southern Asia [2]. However, replication of OPV in humans is frequently accompanied by genetic changes in the vaccine virus. The changes can include reversion of key attenuating mutations and acquisition of other mutations throughout the genome. PV evolves very quickly, partly due to the high error frequency in RNA synthesis: roughly 10^−4^ per base per replication cycle [3]. In addition, recombination contributes to the variability of PV strains [4,5]. Intertypic recombination is a frequent phenomenon in OPV vaccinees, and strains with a recombinant genome have been isolated from both healthy vaccinees and from patients with vaccine-associated poliomyelitis [6–9]. The reversion of the OPV strains to neurovirulence is the underlying mechanism for the rare cases of vaccine-associated paralytic poliomyelitis (VAPP) among OPV recipients and their close contacts, and the occurrence of polio outbreaks associated with circulating vaccine-derived PV (VDPV) [10,11].

The first outbreak of poliomyelitis (21 cases) associated with VDPVs, was reported in 2000–2001, in the Dominican Republic and Haiti [12]. Subsequently, outbreaks due to VDPVs occurred in the Philippines, China, Indonesia, Cambodia, Madagascar, and more recently in Myanmar and Nigeria [13–17]. Prolonged circulation of type 2 VDPVs, responsible for 30 cases of AFP from 1983 to 1993, has been retrospectively demonstrated in Egypt [18]. The duration of VDPV circulation before the outbreaks was estimated through nucleotide divergence to be generally from about 1 to 2.5 years, however, it could reach 10 years in Egypt [16,18,19]. Except in China, all VDPVs implicated in outbreaks were recombinants originating from OPV strains; it was suggested that large parts of the genomic regions encoding the non-structural proteins in these strains were derived from unknown non-polio enterovirus related to HEV-C [11–16,18,20].

In most cases, low vaccine coverage is thought to have allowed the circulation of OPV strains in non-vaccinated children and the subsequent genetic and phenotypic drift of these strains to pathogenic circulating VDPVs [16]. The emergence of epidemic VDPVs threatens the success of the program for global eradication of poliomyelitis. Improved surveillance and vaccine strategies limiting VDPV spread are urgently required. Other than low vaccine coverage, little is known about the viral factors and conditions that favor the emergence and circulation of VDPVs [16,19,21].

In Madagascar, type 2 recombinant VDPVs were identified as the causative agent of five cases of poliomyelitis that occurred from October 2001 to April 2002 [14]. We analyzed the implicated VDPV isolates and compared their sequences with those of co-circulating enteroviruses isolated from healthy children living in the small area where most of the poliomyelitis cases were reported. We demonstrate substantial co-circulation and evolution of HEV-C and OPV strains by recombination resulting in an unexpected genetic diversity. This gives further insight into the characteristics of viral ecosystems, evolution processes and factors that could favor the emergence of pathogenic recombinant VDPVs.

## Results

### Sequence Analysis of the Madagascan VDPV Genomes

**Figure 1:**
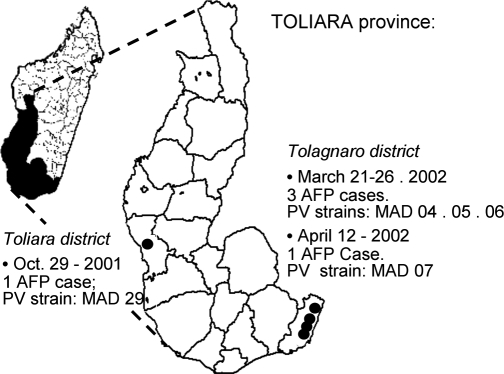
Map of the Toliara Province in Madagascar This map indicates the location of the two districts where the poliomyelitis outbreaks occurred. Names of the PV strains isolated from the patients with AFP are indicated.

Five type 2 PV strains were isolated from stool samples of each of the children with AFP collected in the Toliara (MAD 29 strain) and Tolagnaro (MAD 04, 05, 06, and 07 strains) districts (Figure 1).

Overlapping reverse transcriptase (RT)-PCR products covering the entire genome of each isolate were sequenced (accession numbers for nt sequence data are given below). The nt sequences of MAD 29, isolated in 2001, differed from those of the other strains isolated in 2002. In contrast, the genomes of the strains isolated during 2002 (MAD 04 to MAD 07) were closely related: MAD 04, 05, and 06 exhibited only 4 or 5 nt differences and MAD 07, the most divergent, differed from the three others at 18 to 22 nt positions. Therefore, we focused mainly on MAD 04, considered to be the 2002 prototype strain and on MAD 29.

**Figure 2:**
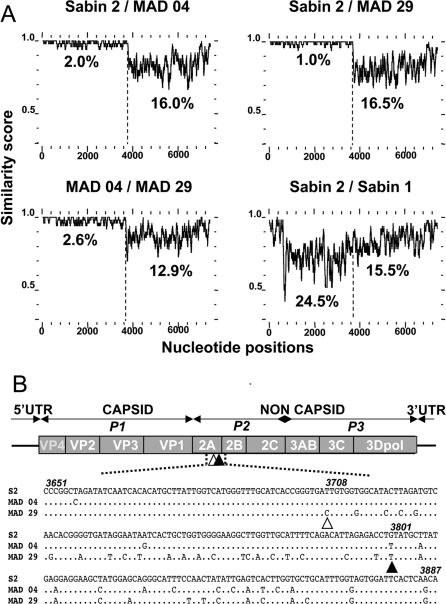
Genomic Features of the Poliovirus Isolates (A) Plots of similarity between a set of aligned genomes (Sabin 2, MAD 04, MAD 29, and Sabin 1), using a 50-nt sliding window. Approximate nucleotide positions in the poliovirus genome are indicated. Average nucleotide identities between the 5′ vaccine part and the 3′ non-vaccine part of the recombinant genomes are shown. (B) Sites of the recombination junctions in the recombinant VDPV genomes. The genetic organization of the PV genome is shown, including the 5′- and 3′-UTR. Genomic regions *P1* to *P3* encoding viral proteins (VP4 to 3Dpol) are indicated. Open and closed triangles indicate the approximate sites of the recombination junctions of the MAD 29 and MAD 04 recombinant genomes, respectively. Both junctions are located at the 3′ end of the protease 2A genomic region. MAD 04 and MAD 29 sequences (nt 3651–3890) on both sides of the recombination junctions are given and compared with Sabin 2 sequences (S2). Nucleotide numbering according to Sabin 2 sequences is given in italics.

The nt sequences of MAD 04, MAD 29, and the Sabin 2 strain were aligned and compared (Figure 2). Two different genomic regions could be clearly distinguished: the 5′-halves of the genomes were all similar and appeared to be derived from Sabin 2 with differences at few positions; in contrast, the 3′-halves of MAD 04 and MAD 29 diverged substantially from that of the Sabin 2 genome.

The genomic region encoding the capsid protein VP1 (903 nt) is widely used in studies of the molecular epidemiology of PV strains and in this region, MAD 04 and MAD 29 differed from Sabin 2 by 23 and 9 substitutions, including 18 and 6 synonymous ones, respectively. Only three of these substitutions were shared by MAD 04 and MAD 29. The general molecular clock of PV has been estimated to be about 1% nt substitutions per year from studies on circulating strains and on viruses excreted from chronically infected immunodeficients [5,22–24]. A more precise estimation relies on the percentage of substitutions per synonymous site. In particular, from the analysis of similar recombinant type 2 VDPVs circulating in Egypt, the evolution rate of the capsid VP1 region appeared to be 2.5 (± 0.7).10^−2^ substitutions per synonymous site and per year [18]. This suggests that VDPVs MAD 04 and MAD 29 had been circulating before isolation for about 22 to 40 mo and 7 to 13 mo, respectively.

The non-vaccine part of MAD 04 and MAD 29 genomes differed substantially from each other and from the Sabin 2 genome (from 12.9% to 16.5% nt; Figure 2). The percentages of nt divergence were similar to those that differentiate the 3′-halves of different PV or HEV-C genomes. For example, the 3′-half of the genome (3,730 nt) of Sabin 2 differs by 18.0% from that of the prototype strain G12 of coxsackievirus A17 (CA17).

The sites of recombination in the genomes of the Madagascan VDPVs, i.e., the junction between the vaccine part and non-vaccine part of their genome, were in the region encoding the C-terminal third of protease 2A (a 149-amino acid protein). According to the sequence alignments presented in Figure 2, the likely sites of recombination in the genome of MAD 04 and other VDPV strains isolated in 2002 (MAD 05 to MAD 07) were all close to nt 3,801 (codon 139 of 2A). The likely site of recombination in MAD 29 was different, and close to nt 3,708 (codon 108 of 2A).

The patterns of nt substitutions in the Sabin 2 regions of the VDPV genomes, the sequences of the non-vaccine genomic regions, and the positions of the recombination sites between these two regions indicated that the PV strains isolated in Madagascar in 2001 (MAD 29) and those isolated in 2002 (MAD 04 to 07) represented two different recombinant VDPV lineages that had emerged and evolved independently.

### Phenotypic Characteristics of the Madagascan VDPVs

The substitutions in the vaccine part of the VDPV genomes are shown in Table 1. Some of them may have affected the phenotype of the vaccine strain Sabin 2. To detect any effect of these substitutions on antigenic structure, MAD 04 and MAD 29 strains were compared to the Sabin 2 strain using a Sabin 2 strain-specific monoclonal antibody (MAb) collection (Table 2) [25,26]. Several MAbs directed against the neutralization antigenic sites 1a and 3a of Sabin 2 had only weak neutralizing effects on both strains MAD 04 and MAD 29. Interestingly all MAbs directed against the antigenic site 2a neutralized MAD 04 and Sabin 2 but did not neutralize MAD 29. In contrast, one MAb (MAb 1108) directed against the neutralization antigenic site 3b showed a weak reactivity against MAD 04 but neutralized both MAD 29 and Sabin 2. Neither MAD 04 nor MAD 29 was neutralized by MAb 1233, highly specific for Sabin 2, and used for intratypic analysis of PV [27]. Thus, the antigenic structures of both VDPVs differed from that of Sabin 2. Furthermore, the two strains exhibited distinct neutralizing epitope maps, in agreement with them representing two different VDPV lineages. The location of amino acid mutations likely to be responsible for the MAb reactivity of both VDPVs is shown in Table 1.

**Table 1:**
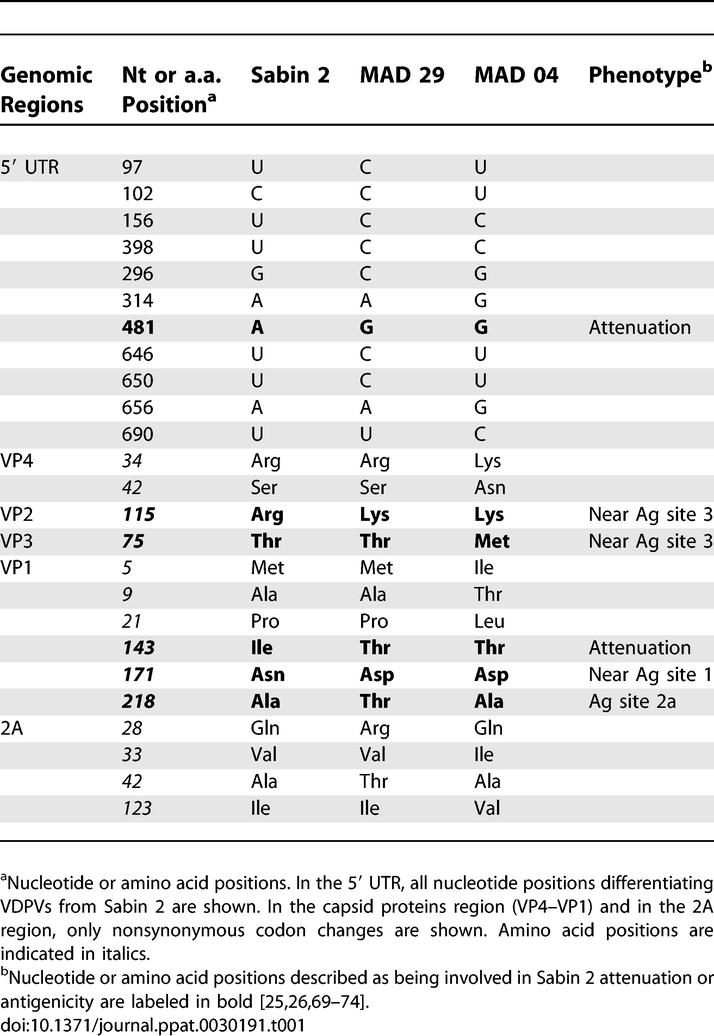
Nucleotide Substitutions and Nonsynonymous Codon Changes in the Vaccine Part of the Genome of the Madagascan VDPVs

**Table 2:**
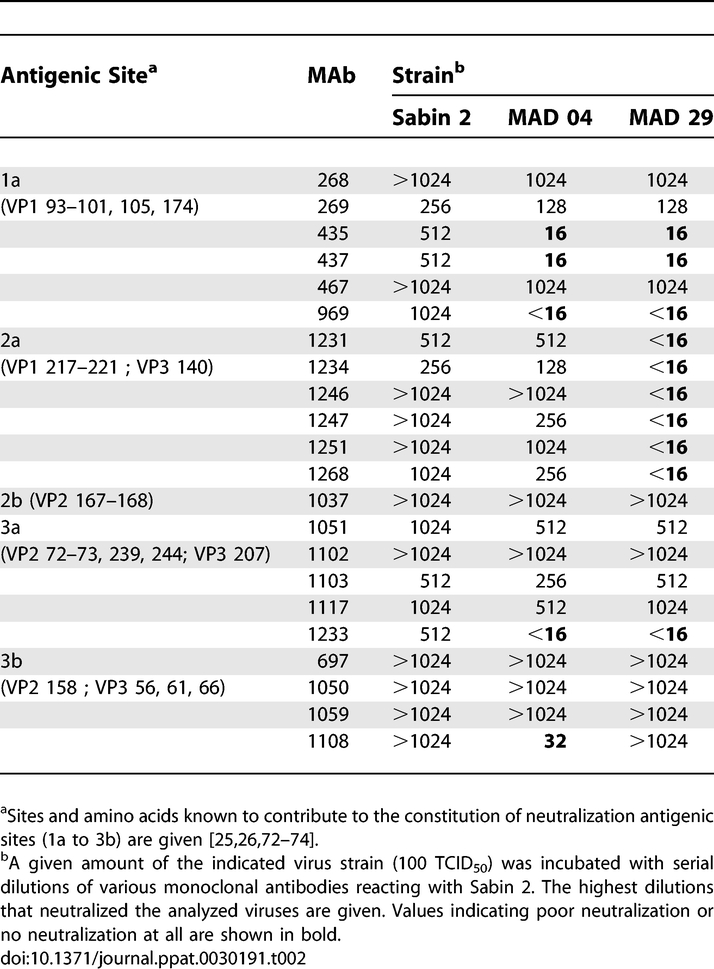
Antigenic Structure of the VDPVs MAD 04 and MAD 29

**Figure 3:**
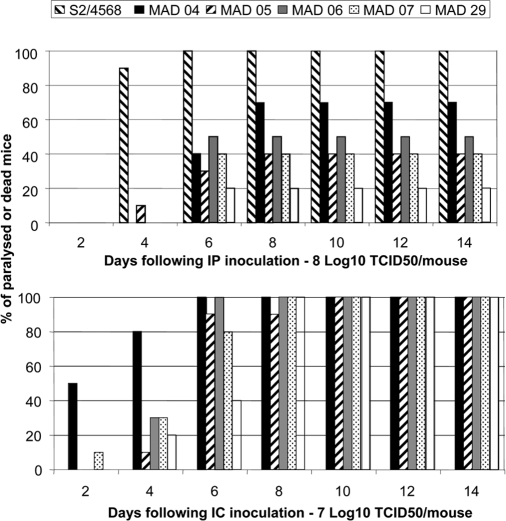
Neurovirulence of VDPVs in Transgenic PVR-Tg Mice A given dose of virus was used to inoculate intracerebrally (IC) or intraperitoneally (IP) groups of PVR-Tg mice expressing the human poliovirus receptor (8–10 animals per virus). Animals were checked daily for 14 d following inoculation for paralysis and death. Percentages of affected mice following inoculation of viruses MAD 04 to MAD 07, MAD 29, and of a positive neurovirulent control virus S2/4568 are given. Strain S2/4568 was used only for IP inoculation. IP or IC inoculation of the vaccine strain Sabin 2 did not induce disease in animals.

The plaque sizes of the VDPV strains isolated in Madagascar were measured (Table 3). The VDPVs produced larger plaques than the Sabin 2 strain (results significant for four of five VDPVs). We also tested the temperature sensitivity of the VDPV by measuring the titers of the viral stocks at optimal (36.0 °C) and supra-optimal (40.2 °C) temperature (RCT test: Table 3). The non-temperature-sensitive and highly neurovirulent type 2 vaccine-derived environmental strain S2/4568 was used as control [28]. Unlike Sabin 2, the difference in titer of all VDPVs and S2/4568 at these two temperatures was small, indicating that they were not temperature-sensitive. We also evaluated the pathogenicity of the Madagascan VDPVs by IC and IP inoculation of groups of homozygous PVR-Tg21 mice [29] with a single virus dose per animal (Figure 3). In contrast to Sabin 2 that did not induce disease, all the Madagascan VDPVs induced paralysis in all IC inoculated animals. They were also all neurovirulent following IP inoculation but the severity of the symptoms differed between strains: strain MAD 04 was the most neurovirulent strain and MAD 29 was the least neurovirulent strain. This was confirmed when the paralytic dose affecting 50% of the inoculated mice (PD_50_) was determined by IC inoculation (Table 3).

**Table 3:**
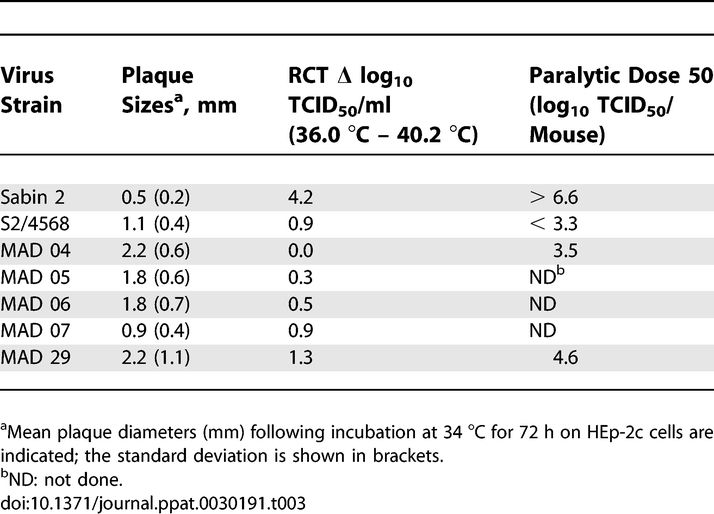
Phenotypic Markers of the VDPVs and Other Poliovirus Strains

The Madagascan VDPVs did not have either the temperature sensitive or attenuated phenotypes that characterize the OPV strains, and therefore appeared to be similar to wild PVs.

### Search for VDPVs and Co-Circulating Enteroviruses in Healthy Children of the Tolagnaro District

Following the VDPV outbreak, two rounds of local vaccination campaigns with trivalent OPV were implemented at 1-mo interval. Two weeks after the second round, 316 stool specimens were collected from healthy children living in the rural villages of the Tolagnaro district to check the effect of these campaigns on the circulation of VDPVs. We also exploited these samples to look for other enteroviruses and to identify putative parents of the unidentified enterovirus sequences present in the VDPV genomes. Healthy children were recruited in the three villages where the AFP cases were reported and in three nearby villages, all located in a swampy coastal area, 2 to 8 kilometers away from each other (Figure 1 and Table 4).

**Table 4:**
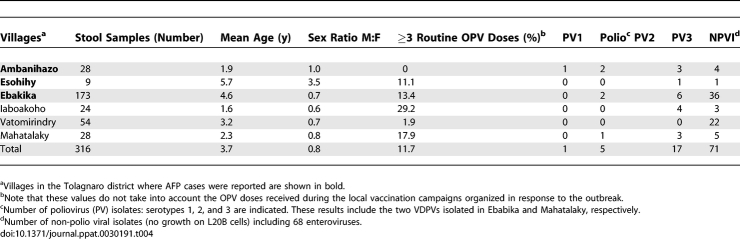
Epidemiological and Virological Data for the Healthy Children

Inoculation of cultured human RD and HEp-2c cells and mouse L20B cells expressing the human PV receptor led to the isolation from these samples of 23 PVs and 71 other viruses inducing cytopathogenic effects only on human cells [30–32] (Table 4). Sixty-eight non-polio viruses were identified as enteroviruses by amplification by RT-PCR of the 5′ UTR regions using nt primers that target highly conserved enterovirus sequences. The 5′ UTR (nt 215–582, according to Sabin 2 nt sequences numbering) and the regions encoding the capsid protein VP1 (nt 3,067–3,376) and the nonstructural proteins 2C (nt 4,123–4,383) and 3D (nt 6,145–6,732) of most of the PV and other enterovirus isolates were sequenced. From the isolation and sequencing data, no evidence of co-infection by different enterovirus serotypes or genotypes was found. However, re-inoculation of cells with positive samples in the presence of neutralizing antibodies directed to the identified virus serotype was not performed to check the possible presence of a second one (see also Discussion section).

Most PV isolates were closely related to one of the original OPV strains with less than 0.5% nt divergence. However, one of the non-recombinant type 2 OPV isolates was more divergent, and the whole VP1 genomic region was sequenced: it showed 0.7% nt divergence from the Sabin 2 sequence (0.5%, in the three other genomic regions), indicating that this strain had been multiplying or circulating for about 8 months. Two PV isolates were OPV type 3 / type 1 intertypic recombinants, as commonly found in vaccinees [6]. Two type 2 isolates were closely related to the recombinant VDPVs of the MAD 04 lineage with less than 1.1% nt divergence in all sequenced genomic regions. Sequencing data of their whole VP1 genomic regions indicated that these new VDPVs (VDPVs 65972 and 68266) were slightly more divergent from the Sabin 2 strain (2.8% and 3.1% nt divergence, respectively) than their AFP counterparts (2.5%). Other than these two recombinant VDPVs, the sequences of the PVs isolated from healthy children provided no evidence of genetic recombination with non-OPV strains.

The nt sequences encoding the VP1 capsid protein of enterovirus isolates contain serotype-specific information [33–35]. We used partial sequencing of the capsid VP1 region to identify 64 enteroviruses [33]. Several echoviruses of the HEV-B species were found including two echoviruses serotype 14 (E14), ten E19, and one E25. Surprisingly, coxsackie A viruses (51 isolates) of the HEV-C species were the most frequent, and they included 12 CA11, 16 CA13, eight CA17, one CA20, and 14 CA24.

These findings indicated the persistence in the Tolagnaro district of VDPV isolates of the MAD 04 lineage despite two rounds of vaccination; they also show that many children were excreting non-poliovirus enteroviruses (21%) in particular HEV-C (16%).

### Genetic Relationship between the VDPVs and the Other Enteroviruses Isolated in the Tolagnaro District

**Figure 4:**
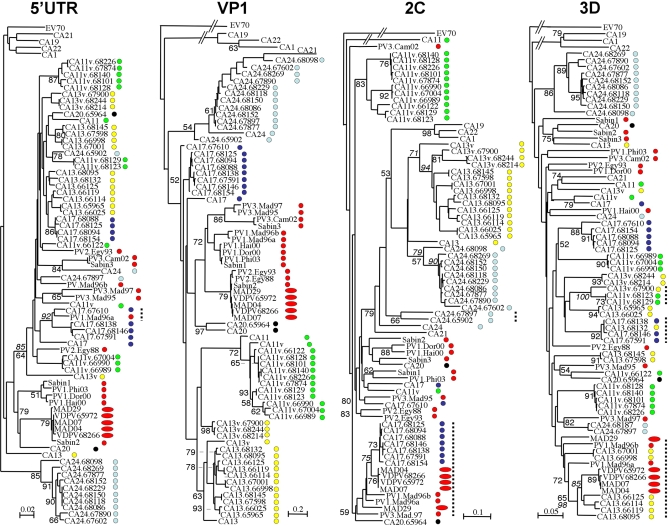
Phylogenetic Trees Depicting Genetic Relationships between Nucleotide Sequences of Enteroviruses of the HEV-C Species These neighbor-joining trees were based on nucleotide sequence alignments of part of the 5′ UTR, the C-terminal third of the VP1-coding regions, part of the 2C-coding regions, and of the 3D^pol^ regions. Branch lengths were calculated using PUZZLE and the Hasegawa, Kishino, and Yano (HKY) model of substitution [63]. The genetic distance is indicated (bar). Numbers at nodes correspond to the percentage of 25,000 puzzle steps supporting the distal cluster; numbers in italics representing the percentage of 1,000 bootstrap pseudoreplicates obtained with the DNADist/Neighbor and SEQBOOT programs of PHYLIP [65] are shown in several cases. Sequences of some of the last wild PV strains (PV3.Mad95, PV1.Mad96a, PV1.Mad96b, and PV3.Mad97) isolated in Madagascar (1995–1997) were used in addition to HEV-C prototype strains (CA1 to CA24). Selected PV strains isolated during the last poliomyelitis outbreak in Finland (PV3.Fin84) and during the recent VDPV outbreaks in Egypt (PV2.Egy93; PV2.Egy88), Haiti (PV1.Hai00), Dominican Republic (PV1.Dor00), and the Philippines (PV1.Phi01) are included. Sequences of the recombinant type 3 PV isolated from a poliomyelitis case in Cambodia in 2002 (PV3.Cam02) were also used. Nucleotide sequences of echovirus 70 (EV70) were used as outgroup. Identifiers of the HEV-C coxsackie A viruses isolated from healthy children in 2002 in Madagascar consist of the given serotype as assessed from partial sequencing [33] and the laboratory number. New VDPV isolates from healthy children are indicated with their laboratory numbers. Spots of different colors differentiate HEV-C serotypes and polioviruses (red). VDPVs from Madagascar are indicated by oblong spots. According to the Eight Report of the ICTV [56], former CA15 and CA18 are considered as antigenic variants of CA11 and CA13, and are now named CA11v and CA13v, respectively. All field isolates from Madagascar, except isolates CA24.65902 and CA24.68187, were sequenced in the four regions. Vertical dotted lines indicate groups of isolates of particular interest that are discussed in the text.

The various enterovirus sequences were aligned and compared in phylogenetic trees. The phylogenetic relationships between the Madagascan HEV-C field isolates, their prototype strains, OPV strains, and the VDPVs isolated in the island and in other countries are shown in Figure 4. The four last wild PV strains isolated in Madagascar, two type 1 strains (PV1.Mad96a and PV1.Mad96b) isolated in 1996 and two type 3 strains (PV3.Mad95 and PV3.Mad97) isolated in 1995 and 1997, respectively, were included. For phylogenetic trees, similar assignments for reconstruction of major clusters and, in most cases, of sub-clusters were obtained using both the maximum likelihood and the genetic distance matrix / neighbor-joining methods.

As expected, the sequences of the two VDPVs isolated from healthy children (VDPVs 65972 and 68266) clustered with those of the VDPVs MAD 04 and MAD 07 in the four phylogenetic trees (Figure 4) confirming that they belong to the same recombinant lineage.

According to VP1 sequences, HEV-C isolates displayed different patterns of genetic diversity depending on their serotype. CA13 and CA11 isolates appeared to be relatively heterogeneous: 83 to 100% VP1 nt identity within their respective clusters, and different sub-clusters could be observed. VP1 sequences of most CA17 and CA24 isolates were more homogeneous with 91%–98% nt identity within their respective clusters. However, isolates CA17.67610, CA24.65902, and CA24.68098 showed only 83%–85%, 72%–75%, and 75%–88% nt identity with their respective counterparts. Therefore, the VP1 nt sequences of these coxsackie A virus isolates showed a relatively high genetic diversity within each of their respective serotypes.

In contrast to VP1 sequences, the nt sequences of the N-terminal part of protein 2C did not necessarily segregate according to the serotype of the isolate**.** The 2C sequences of the VDPVs of the MAD 04 lineage segregated neither with those of the Sabin 2 strain nor with those of other OPV strain serotypes but with those of the major group of CA17 field isolates (92%–94% nt identities; high reliability values). This result strongly suggests that a MAD 04 VDPV ancestor has acquired its non-OPV 2C sequences from a co-circulating CA17 isolate by genetic recombination or that both viruses acquired these sequences from a common ancestor virus. These VDPVs also showed relatively high nt identities (91%–93%) with the wild PV1.Mad96a and appeared related to a certain extent to the two other wild PV1.Mad96b and PV3.Mad97 (86%–90% nt identities) and to the other VDPV MAD 29 (86%–87% nt identities). No other major change appeared in the groupings of the 2C phylogenetic tree compared to the groupings of the VP1 tree, except for the isolate CA24.67897 whose 2C sequences were distantly related to those of the other field CA24 isolates (labeled by dotted lines in Figure 4) indicating that this isolate had acquired its 2C sequences by intratypic recombination.

There were major differences between the groupings of isolates in the phylogenetic tree of the 3D nt sequences and those in the VP1 and 2C trees (Figure 4). The 3D sequences of many isolates, in particular those of the CA11, CA13, and CA17 field isolates, appeared to be split into distantly related clusters. VDPVs of the MAD 04 lineage showed 3D sequences closely related to those of four CA13 isolates (92%–93% nt identities; high reliability values). They were related to PV1.Mad96a (90% nt identity) and to a certain extent to two CA13, to PV1.Mad96b and to VDPV MAD 29 (87%–88% nt identities). The other CA13 isolates and all CA17 isolates were more distant (81%–85% nt identities). Recent intertypic recombination events could be inferred. In particular, the cluster containing CA13.68132, CA17.68138, CA17.68146, and CA17.67591 (99% nt identities; high reliability value), and that with CA13v.67900, CA11v.68123, and CA11v.68129 (94% nt identities) indicate genetic exchanges between CA13 and CA17 isolates and between CA11 and CA13 isolates.

According to the phylogenetic tree built with the 5′ UTR nt sequences, the isolates belonging to each of the major serotypes (CA11, CA13, CA17, and CA24) were in different clusters. This indicates that the 5′ UTR of these serotypes has frequently been subject to recombination.

In order to estimate the number of different recombinant lineages among the Madagascan HEV-C isolates, we systematically considered incongruences between the four different phylogenetic trees (Figure 4) that were supported by reliable values and/or observed using both the maximum likelihood and the genetic distance matrix/neighbor-joining methods. 19 HEV-C recombinant lineages differing by one of the sequenced fragments could be distinguished. In most cases, a good correlation was found between recombinant lineages and groups or subgroups of nt sequences observed in a single tree (as mentioned above for the VP1 tree). Different serotypes and in some cases different recombinant lineages belonging to the same serotype were found in the same village. Moreover, a given recombinant lineage could be isolated in two different villages.

We compared the polypeptides encoded: the peptide relationships did not necessarily parallel the nt relationships. The 2C-polypeptides (87 residues) of the Madagascan VDPVs of the MAD 04 lineage differed from those of the closely related CA17 field isolates at one to two amino acid positions. They were strictly identical to those of PV1.Mad96a and PV1.Mad96b, to those of some other CA17 and CA11 isolates and even to those of some VDPVs isolated in other countries but different from the 2C peptide sequences of other field isolates (16 to 17 amino acids differences for the CA13 field isolates). These results are in good agreement with those of Brown et al. [36] indicating that the 2C peptide sequences of prototypes CA17, CA11, and CA20 are particularly similar to those of PVs. As expected, the peptide sequences of the 3D regions (196 residues) are highly conserved for all serotypes and even the 3D amino acid sequences of the prototype strains CA13 and CA24, as well as those of some HEV-C field isolates or those of the wild PV1.MAd96a are strictly identical. The 3D polypeptides of the MAD 04 lineage differed from those of the four most closely-related CA13 field isolates at two to five amino acid positions, those of their most divergent HEV-C field isolates (nt level) showing no more than six different amino acids. These results confirmed that viral 2C and particularly 3D HEV-C polypeptides are highly conserved. They are probably poorly permissive to most amino acid modifications acquired by mutations or recombination. The corollary of this is that conserved peptidic sequences could favor genetic exchanges.

Although the genetic exchanges between these enteroviruses appeared to have a limited effect on the variability of the peptide sequences, our findings indicated a substantial genetic diversity of HEV-C isolates in the Tolagnaro district due to nt divergence as well as to intertypic and interspecific recombination involving PV. As expected, phylogenetic analysis of the nt sequences of the HEV-B isolated in Madagascar yielded evidence of interspecific recombination events neither between the VDPVs and the three serotypes of HEV-B isolates (Figure S1) nor between HEV-B and HEV-C isolates (not shown).

## Discussion

Here, we describe two different lineages of type 2 PV/HEV-C recombinant VDPVs that appeared and circulated independently in Madagascar, and that induced paralytic poliomyelitis. The search for enterovirus circulating in the small area where most of the poliomyelitis cases occurred indicated an unexpectedly high diversity of coxsackie A viruses belonging to HEV-C that co-evolved by intertypic and interspecific recombination involving PVs.

Circulation of endemic type 2 VDPVs and wild strains associated with low vaccine coverage in Egypt from 1983 to 1993 has been described [18]. The isolates appeared to be derived from a single OPV infection and all belong to the same lineage. The specificities of the outbreak we describe are that it occurred in a country where wild PV strains were eliminated by 1997 and that it was due to two different type 2 VDPV lineages.

Studies that established the rates of infection and indirect immunity against PV in contacts of vaccinees indicate that the Sabin 2 strain spreads to unvaccinated children more easily than type 1 and 3 vaccine viruses [37–39]. Sabin 2 is highly immunogenic in vaccinees and induces higher seroconversion rates than do the other two OPV serotypes [38,40,41]. Nevertheless, the Sabin 2 strain also has the bad reputation of inducing the highest rate of VAPP following contact with vaccinees [9,42]. These characteristics, may explain why Sabin 2-derived viruses can in some circumstances, circulate in human populations and subsequently acquire pathogenic characteristics [43]. The discovery of two different lineages of type 2 VDPVs in Madagascar confirms this notion. Furthermore, new lineages of type 2 VDPVs were isolated from AFP cases in the southern part of Madagascar in 2005 (Rakoto Andrianarivelo et al*.* unpublished data and [44]). Very recently, numerous poliomyelitis cases due to different type 2 VDPV lineages were reported in Nigeria, a country still endemic for wild PVs [17,45].

We report two VDPV isolates genetically similar to the MAD 04 lineage and isolated 2 months after the last AFP case and following two rounds of local vaccination campaigns; this indicates that this type 2 VDPV lineage was widespread and well established in the population and has not been cleared by these campaigns. However, two weeks after the second local round of vaccination only 7% of children were found to excrete Sabin-like PVs. Previous immunisation responses may have limited subsequent multiplication and excretion of vaccine strains, and numbers of excretors [39]. However, we cannot exclude the possibility that interference between OPV strains and the numerous endogenous enteroviruses circulating in the province, or some other host and environmental factors contributed to reducing vaccine strain multiplication and to limiting OPV immunogenicity [40,41]. Unfortunately, no data concerning OPV responses following the local vaccination campaigns were available. However, no further AFP cases due to this VDPV lineage have been reported in the area. This lineage is thought to have disappeared, possibly after the two national rounds of vaccination (“national immunization days”) that followed in September and October 2002.

The characterization of the enteroviruses co-circulating with the MAD 04 lineage in the Tolagnaro district indicated that both the frequency and diversity of HEV-C were substantial. Five of the six HEV-C serotypes known to grow in cultured cells were found (three serotypes can only be isolated in new-born mice) [36]. Moreover, the genetic diversity within each serotypes was high due to nt sequences divergence and number of subgroups distinguished and to frequent recombination events involving all four sequenced regions (19 different recombinant lineages differing by one sequenced fragment). Similar evolution processes, involving frequent intertypic recombination, have already been described mostly for HEV-B [46–52] and PVs [5,8,53,54]. In this study we show that recombination also contributes considerably to the genotypic diversity of HEV-C. Different HEV-C serotypes circulate in Cambodia and a previous study has pointed the relatively high frequency and the wide distribution of HEV-C in different regions of Madagascar [32,53]. However, this is the first time that such a high frequency and diversity of HEV-C isolates, and, in general, of isolates of the same RNA virus species, have been described in such a small area (about 25 × 10 Km) and in such a small population (316 children). Our observations thus shed a new light on the characteristics of viral ecosystems and their evolution.

Most VDPV strains described as OPV/HEV-C recombinants were isolated in countries in which wild PVs have been eradicated, so the unidentified human enterovirus has been presumed to be HEV-C [ 11,12,14–16,18,20]. Up to now only a Sabin3/HEV-C recombinant from Cambodia was shown to be directly related to indigenous CA17 strains in the 2BC genomic region and to a CA13 isolate in the 3D region [53]. Here we show that recombinant VDPVs are similarly closely related to CA17 isolates in the 2C region and to CA13 isolates in the 3D region. These findings indicate that CA17 and CA13 isolates are particularly suitable partners for sharing nt sequences with PVs by means of recombination. Moreover, this study indicates for the first time that HEV-C isolates sharing sequences closely related to a VDPV lineage were co-circulating with this lineage in the very place where the outbreak occurred. These observations lend considerable support to the idea that there is frequent genetic recombination between OPV strains and at least some HEV-C serotypes, and that this plays a role in the emergence and/or evolution of VDPVs.

Despite the recombinant OPV/HEV-C features of most VDPVs, field isolates with the capsid of HEV-C and non-structural parts from OPV were not found in this study. To our knowledge such recombinants have not been described elsewhere. The viral replication machinery or selection factors that are known to act in vivo to shape the features of intertypic OPV recombinant genomes [6] may exclude such HEV-C/OPV recombinants. This hypothesis was recently supported experimentally with recombinant viruses generated from PVs and CA20 or CA21 prototype strains [55].

Genetic recombination requires the co-infection of the host and cells by at least two parental viruses. In fact, search for viral mixtures was not the primary goal of the study and the viral isolation procedures were poorly adapted to detect them. However, recent inoculation of HEp-2c cells and RD cells with almost all PV positive samples, in the presence of a mixture of neutralizing antipoliovirus antibodies, indicated that about 25% of these samples contain at least one other enterovirus serotype that was masked on L20B cells during PV isolation (not shown).

It is interesting to note that the nt sequences of the CA17 and the CA13 isolates that are closely related to those of the MAD 04 lineage are also related to a certain degree to the sequences of the VDPV MAD 29 and, despite at least 5 y of nt drift, remain related to sequences of wild PVs isolated in 1996 and 1997. Relationship between these isolates probably results from co-circulation of these viruses and evolution by recombination since many years throughout the Tolagnaro Province. This suggests that PVs and HEV-C have been occupying the same or at least overlapping viral ecological niches, both species contributing to evolution and the generation of diversity by intratypic, intertypic and interspecific recombination. These various considerations argue for a long-term evolution process involving wild or vaccine PVs and some HEV-C viruses (at least CA17 and CA13) and lend support to the proposal that PVs and HEV-C should be considered to be members of a single species [5,36,55,56].

The functional role of interspecific genetic exchange in the evolution of PV/HEV-C recombinants is still unclear. Type 1 non-recombinant and vaccine/vaccine recombinant VDPVs have circulated for about one year in China and in Romania, respectively, showing that recombination with HEV-C is not essential for OPV strains to become circulating VDPVs [13,57]. Nevertheless, recombination frequency suggests that genetic exchanges may allow PV and HEV-C to evolve rapidly and to acquire some functions like those that are necessary for efficient circulation in the population. Although less likely we cannot exclude that interspecific recombination may be a neutral phenomenon and simply testify that OPV and HEV-C strains are well established in the population thereby increasing considerably the frequency of encounters and recombination. Indeed, the frequency of HEV-C circulation is high in both Madagascar and Cambodia, countries in hot humid tropical zones [32,53]. Possibly, the climate along with sanitation and hygiene are important risk factors for VDPVs and enterovirus spread. It is also plausible that particular physiological, immunological and genetic factors in the local human population help these viruses circulate and co-evolve.

To our knowledge, the observed HEV-C frequency and biodiversity and the simultaneous presence of VDPVs in such small areas and such small human populations have not previously been described. This biodiversity combined with the poor polio vaccine coverage, may make the local ecosystem a “cauldron” particularly favorable for the emergence of new recombinant VDPVs and possibly new pathogenic coxsackie A virus strains. This argues strongly for an increased surveillance in such areas and for the continuation of studies to elucidate the viral, human and environmental factors that shape viral genetic diversity and contribute to the emergence of VDPVs.

## Materials and Methods

### Cells and viruses.

Human HEp-2c, RD cells, and murine L20B cells (murine L cells expressing the PV human receptor [31]) were grown as monolayers in Dulbecco's modified Eagle medium supplemented with 5% fetal calf serum.

The poliovaccine viruses, Sabin 1, 2 and 3 were obtained from the WHO [Behringwerke (S0+1)] “master seeds”. The second passage at 34 °C in HEp-2c cells of the original seed was used to prepare viral stocks.

VDPVs MAD 04 to MAD 07 and MAD 29 were isolated on human RD and murine L20B cells from specimens (stools) from poliomyelitis cases according to WHO recommendations for poliomyelitis surveillance.

PV strain S2/4568 has been described previously: it is a highly neurovirulent and non-temperature sensitive vaccine-derived strain, of serotype 2 [28].

### Field investigations.

To determine the frequency and circulation of VDPVs field investigations were conducted on June 21 and 22 in rural villages of the district of Tolagnaro. 316 stool specimens were collected among healthy children. A baseline questionnaire, which included date of birth, sex, site of enrolment, and previous routine immunization based on health cards, was completed for each child. This investigation was conducted as part of the national AFP surveillance recommended by WHO for poliomyelitis surveillance purposes. It was organized with the agreement and help of the Madagascan Ministry of Health and Family planning and biological materials were collected after obtaining informed consent from the parents.

### Viral isolation.

Extracts of stool specimens were treated with chloroform and used to inoculate RD and HEp-2c cell lines, for enterovirus isolation, and L20B cells. All L20B PV isolates were characterized using microneutralization serotyping tests [30]. Isolates showing cytopathogenic effects only on HEp-2c or RD cell lines were considered to be non-polioviruses and analyzed further by molecular typing (see below).

Poliovirus isolates were analyzed by multiple restriction fragment length polymorphism assays involving amplification of two regions of the genome (the VP3/VP1 capsid region and the 3D^pol^-3′-UTR) and the restriction enzymes DpnII, DdeI, HinfI and RsaI as previously described [58]. Strains identified by this method as mutant and recombinant PV vaccine strains were further analyzed by partial sequencing.

### RT-PCR.

Viral RNA was reverse transcribed as described previously either directly from viral stocks, following heat denaturation of the virions [8], or following viral RNA extraction [49]. DNA fragments were amplified by PCR as described by Chevaliez et al. [49] using previously described primers [8,33,49]. Depending on the presence of a single or multiple bands in the gel, pooled PCR products (100 μl) were either directly purified using the QIAquick PCR Purification kit (Qiagen) or excised from agarose gel following electrophoresis and purified with the QIAquick Gel Extraction kit (Qiagen).

The 5′-end of the viral genome was amplified with the 5′/3′ RACE kit (Roche), as described in the manufacturer's protocol. Briefly, viral RNA was used for first-strand cDNA synthesis using the primer UC52 [8]; the cDNA was purified and a dA-tailing reaction was carried out. We then amplified the dA-tailed cDNA by PCR using the primer UC52 and oligo-dT.

### Sequencing.

The amplified DNA fragments were directly sequenced using the Big-Dye Terminator Cycle Sequencing Ready Reaction Kit on the ABI Prism DNA 377 Sequencer (Perkin-Elmer Applied Biosystems) according to the manufacturer's protocol and using primers described in Guillot et al. and Caro et al. [8,33].

Alternatively, conditions for RT-PCR amplification and cycle sequencing were as described previously [18], using the primers listed in Yang et al. and Kew et al. [12,18]. Sequencing was performed in both directions, and every nt position was sequenced at least once on each strand.

### Molecular typing of enteroviruses.

A fragment of 299 to 322 bp corresponding to the 3′ third of VP1 capsid was compared with the corresponding region of available prototype sequences, using the CLUSTAL W alignment program [59]. The GenBank database was also screened for similar sequences using the FASTA program [60]. Scores were established for each strain according to nt identity and amino acid similarity with the closest prototype strains. The serotype of the field isolates was assumed to be that of the closest prototype strain according to the results of pairwise comparisons of nt sequences, as previously described [33]. In most cases, nt identities with the homologous prototype strains were higher than 75% [33]. However, for many CA24 isolates, nt identities were between 71% and 75%. In this case the putative serotype was supported by the serotype associated with the most similar enterovirus nt sequences present in data banks, usually giving higher nt identities (>75%). Coxsackie A viruses serotypes 15 and 18 are now considered as antigenic variants of coxsackie A virus serotype 11 and 13 and are named in this work CA11v and CA13v, respectively [56].

### Alignment of sequences and phylogenetic analysis.

Phylogenetic relationships between strains were established by comparing the sequences determined and aligning them with those of other known human enteroviruses, using the alignment program ClustaL W or Clustal X [59,61]. The degree of nt sequence identity and of protein similarity between strains was determined using the default scoring matrices.

Complete genome sequences were compared following alignments with the plot-similarity program of GCG version 10.1 software (Genetics Computer Group, Madison, Wisconsin), using a 50-nt sliding window and the default scoring matrix [62].

Phylogenetic relationships between sequences were inferred by the maximum likelihood method with PUZZLE 4.0, which uses QUARTET PUZZLING as the tree search algorithm [63]. The Hasegawa, Kishino, and Yano (HKY) model of substitution for nt with a Ts/Tv of 8.0 was used [64]. Trees were constructed using neighbor-joining of PHYLIP (Phylogeny Inference Package) version 3.6 [65] and branch length given by Puzzle. The reliability of tree topology was estimated using 25,000 puzzle steps.

Alternatively, phylogenetic relationships were inferred by DNADist/Neighbor of PHYLIP [65] and a genetic distance matrix was calculated using the F84 model of nt substitution with a transition/transversion ratio (Ts/Tv) of 8.0. The robustness of phylogenies was estimated by bootstrap analyses with 1000 pseudoreplicate data sets generated with the SEQBOOT program.

Trees were drawn with the TREEVIEW [66] or NJ Plot programs [67].

### Temperature sensitivity.

The temperature sensitivity of viruses was evaluated by studying the reproductive capacity of each virus strain at various temperatures (standard RCT test). RCT is defined as the difference between the log_10_ virus titer of a viral stock measured at 36.0°C and that at 40.2°C. Titers were determined on RD cells by an endpoint micromethod after 5 days of incubation at the appropriate temperatures, and are expressed in TCID_50_ per ml [68]. Viruses with RCT values above 2 were considered to be temperature sensitive. See also Supporting Information for details (Protocol S1).

### Virus plaque sizes.

Plaque diameter was determined by using virus-infected HEp-2c cells maintained under a 0.9% agarose overlay and stained after 3 days of incubation at 34 °C, in a 4% CO2 incubator. For each virus, the diameter of all isolated plaques (about 30 plaques) was measured and mean plaque diameter and standard deviation were calculated (Protocol S1).

### Antigenic structure analysis.

The antigenic properties of viruses were studied by a microneutralization assay as previously described [25,26] using PV Sabin-specific MAbs corresponding to antigenic sites 1 to 3. One hundred TCID_50_ of the challenge virus were used in the test.

### Assay of neurovirulence in PVR-Tg mice.

Viruses were tested for neurovirulence in homozygous PVR-Tg21 mice which are susceptible to PV infection (generous gift from A. Nomoto) [29]. Groups of ten PVR-Tg21 mice (5 males and 5 females) were inoculated intracerebrally (ic; 40 μl) and intraperitoneally (ip; 1 ml) with a single dose of virus (7 and 8 Log10 TCID_50_/mouse, respectively). Challenged mice were monitored daily for 14 days and clinical symptoms (paresis, paralysis, or death) recorded for each mouse.

For evaluating the viral dose that induced paralysis or death in 50% of mice (PD_50_) groups of eight (4 males and 4 females) 5- to 6-week-old mice were used. Animals were inoculated (IC) with 40 μl of tenfold dilutions in Dulbecco's modified Eagle medium of virus stocks containing 0.1% fetal calf serum. Mice were inoculated to cover the viral titer range, causing disease in 100 to 0% of mice. In some cases, although the viral suspension with the highest or lowest titer available was used, the 100% or 0% paralytogenic dose could not be attained. To confirm the inoculated dose, viral suspensions were back-titrated after inoculation. Inoculated mice were monitored for 21 days and the PD_50_ was calculated by the method of Reed and Muench [68]. All experiments were conducted in full compliance with French regulations regarding laboratory animal welfare. Protocols were approved by the Veterinary Staff of the Central Animal Facility of Institut Pasteur.

## Supporting Information

Figure S1 Phylogenetic Trees Depicting Genetic Relationships between Nucleotide Sequences of Enteroviruses of the HEV-B Species and PVs These neighbor-joining trees were based on nucleotide sequence alignments of different part of the genomes and built as described for Figure 4. Sequences of selected HEV-B, echovirus prototype strains (E14, E19, and E25) are included.
(1.5 MB PDF)

Protocol S1 Temperature Sensitivity and Viral Plaque Sizes (46 KB DOC)

### Accession Numbers

The nt sequence data reported in this article are available from the EMBL GenBank under accession numbers AM084223-AM084225 and AM884184-AM884185 (VDPV complete genomes). Partial genomic sequences are also available: AM774327-AM774334 (other VDPVs), AM774339-AM774354 (wild PVs), AM778603–778661, AM779098–779160, AM774410, AM779258-AM779316, and AM779413-AM779471 (HEV-B and HEV-C isolates). The accession numbers for other sequences used in phylogenetic trees are D00820 (EV70), AY302440 (E14), AY302544 (E19), AY302549 (E25), AF499635 (CA1), AF499636 (CA11), AF499638 (CA11v), AF499637 (CA13), AF499640 (CA13v), AF499639 (CA17), AF499641 (CA19), AF499642 (CA20), AF546702 (CA21), AF499643 (CA22), D90457 (CA24), AF448782 (PV2.Egy88), AF448783 (PV2.Egy93), AF405669 (PV1.Hai00), AF405690 (PV1.Dor00), AB180070 (PV1.Phi03), AB205395 (PV3.Cam02).

## Abbreviations

AFP: acute flaccid paralysis
CA: coxsackie A virus
HEV-C: human species C enterovirus
MAb: monoclonal antibody
OPV: oral poliovaccine
PV: poliovirus
TCID_50_: 50% tissue culture infective dose unit
VAPP: vaccine-associated paralytic poliomyelitis
VDPV: vaccine-derived poliovirus
