# Supplementary material for: Co-Circulation and Evolution of Polioviruses and Species C Enteroviruses in a District of Madagascar
Source: PLoS Pathog. 2007 Dec 14;3(12):e191. doi: 10.1371/journal.ppat.0030191 (PMC2134956; doi:10.1371/journal.ppat.0030191)
Supplement: Supplementary file 1 [file ppat.0030191.sg001.pdf]

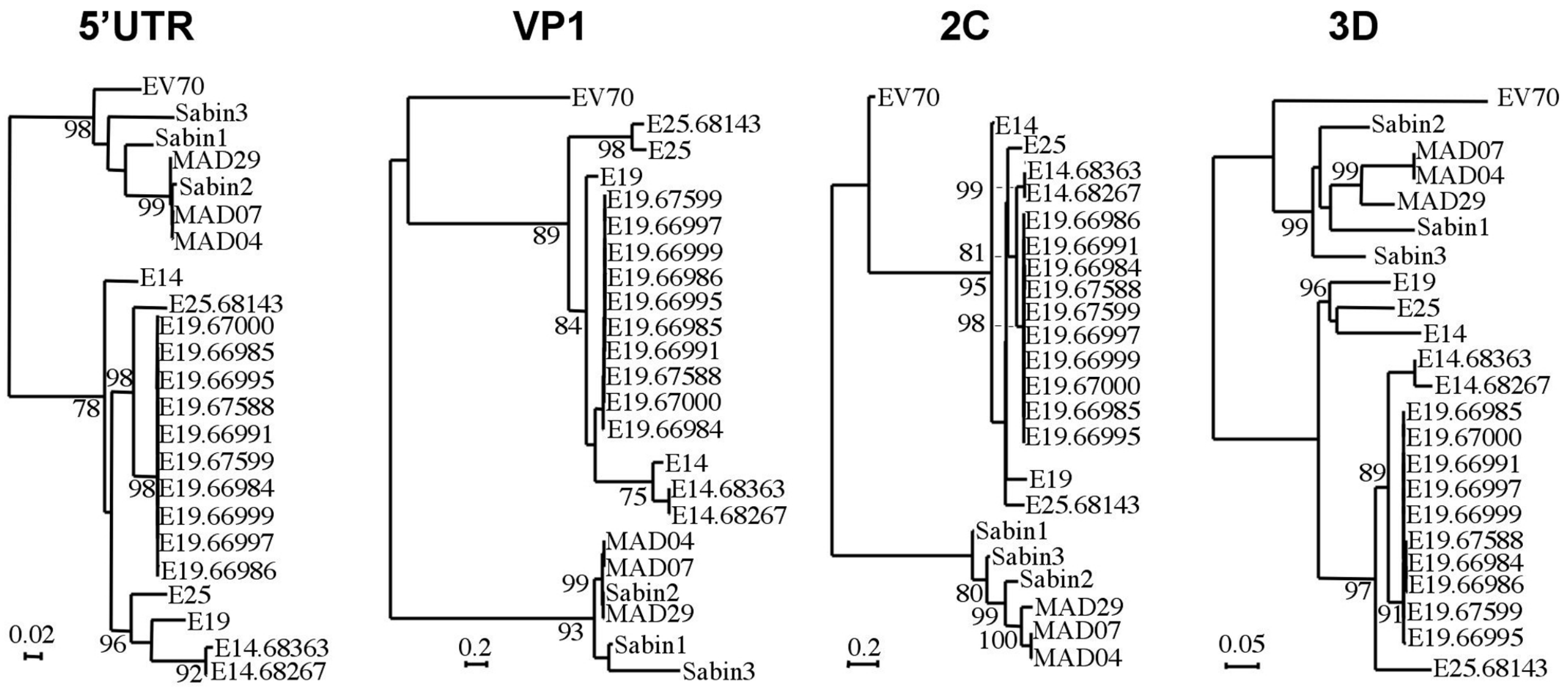

5'UTR
EV70
Sabin3
Sabin1
MAD29
Sabin2
MAD07
MAD04
E14
E25.68143
E19.67000
E19.66985
E19.66995
E19.67588
E19.66991
E19.67599
E19.66984
E19.66999
E19.66997
E19.66986
E25
E19
E14.68363
E14.68267
98
99
98
78
98
96
92
0.02
VP1
EV70
E25.68143
E25
E19
E19.67599
E19.66997
E19.66999
E19.66986
E19.66995
E19.66985
E19.66991
E19.67588
E19.67000
E19.66984
E14
E14.68363
E14.68267
MAD04
MAD07
Sabin2
MAD29
Sabin1
Sabin3
98
89
84
75
99
93
0.2
2C
EV70
E14
E25
E14.68363
E14.68267
E19.66986
E19.66991
E19.66984
E19.67588
E19.67599
E19.66997
E19.66999
E19.67000
E19.66985
E19.66995
E19
E25.68143
Sabin1
Sabin3
Sabin2
MAD29
MAD07
MAD04
99
81
95
98
80
99
100
0.2
3D
EV70
Sabin2
MAD07
MAD04
MAD29
Sabin1
Sabin3
E19
E25
E14
E14.68363
E14.68267
E19.66985
E19.67000
E19.66991
E19.66997
E19.66999
E19.67588
E19.66984
E19.66986
E19.67599
E19.66995
E25.68143
99
99
96
89
97
91
0.05
