# Supplementary material for: Co-Circulation and Evolution of Polioviruses and Species C Enteroviruses in a District of Madagascar
Source: PLoS Pathog. 2007 Dec 14;3(12):e191. doi: 10.1371/journal.ppat.0030191 (PMC2134956; doi:10.1371/journal.ppat.0030191)
Supplement: Supplementary file 2 [file ppat.0030191.sd001.doc]

**Protocol S1**

**Temperature sensitivity**

The temperature sensitivity of viruses was evaluated by studying the reproductive capacity of each virus strain at various temperatures (standard RCT test). Each virus stock, with a titer of 8.7 ± 0.5 log_10_ 50% tissue culture infective dose units per ml (TCID_50_/ml), was decimally diluted through 10-8 in Dulbecco’s modified Eagle medium. A 0.05 ml volume of each virus dilution was inoculated into 10 wells of each of two 96 wells plastic plates. A 0.15 ml volume of medium supplemented with 3% fetal calf serum and containing 2 x 10^4^ HEp-2c cells was then added to each well. One plate was incubated at 36.0°C (optimal temperature) in an incubator, and the second was submerged in a waterbath at 40.2°C (supraoptimal temperature). After 5 days of incubation cells were stained and the titer (TCID_50_/ml) was calculated for each temperature using the Reed and Muench method [68]. RCT is defined as the difference between the log_10_ virus titer of a viral stock measured at 36.0°C and that at 40.2°C. Titers were determined on HEp-2c cells by an endpoint micromethod after 5 days of incubation at the appropriate temperatures, and are expressed in TCID_50_ per ml [68]. Viruses with RCT values above 2 were considered to be temperature sensitive.

**Virus plaque sizes**

Six well plastic plates were seeded with 2 x 106 HEp-2c cells per well in Dulbecco’s modified Eagle medium supplemented with 5% fetal calf serum and kept in an incubator at 37°C and 5% CO2 for about 24 hours. Cells monolayers were washed once with medium without serum, inoculated with 0.4 ml per well of decimally diluted viral stocks (2 wells per dilution) and incubated at 37°C for 30 min, with manual shacking every 10 min. The viral inoculum was removed and replaced by 3ml of a mixture containing 1 volume of 1.8% agarose (SIGMA) in water (kept molten at 45°C) and 1 volume of Dulbecco’s Minimal Essential Medium 2X (maintained at 37°C) supplemented with L-glutamin, 4% fetal calf serum, MgCl2 50mM, 4% Tricin 1M pH7.4, Bicarbonate 2.2 g/l, penicillin and streptomycin. After 30 min at room temperature plaques were incubated upside down at 34°C in a 5% CO2 incubator for 3 days. Agarose containing medium was then removed, cells were stained and isolated viral plaques (> 30) were measured. Mean plaque diameter and standard deviation were calculated.
